# Influence of pyrolysis temperature and production unit on formation of selected PAHs, oxy-PAHs, N-PACs, PCDDs, and PCDFs in biochar—a screening study

**DOI:** 10.1007/s11356-017-0612-z

**Published:** 2017-11-08

**Authors:** Eva Weidemann, Wolfram Buss, Mar Edo, Ondřej Mašek, Stina Jansson

**Affiliations:** 1 Department of Chemistry, Umeå University, SE-901 87 Umeå, Sweden; 2 UK Biochar Research Centre, School of GeoSciences, University of Edinburgh, Edinburgh, UK

**Keywords:** Polychlorinated dibenzo-*p*-dioxin, Polychlorinated dibenzofuran, Polycyclic aromatic hydrocarbons, Oxygenated polycyclic aromatic hydrocarbons, Nitrogen-containing polycyclic aromatic compounds

## Abstract

The influence of reactor type and operating conditions of the pyrolysis unit on the final concentration of toxic contaminants in biochar remains unclear. Therefore, we determined the concentrations of polycyclic aromatic hydrocarbons (PAHs), oxygenated polycyclic aromatic hydrocarbons (oxy-PAHs), nitrogen-containing polycyclic aromatic compounds (N-PACs), polychlorinated dibenzo-*p*-dioxins (PCDDs), and dibenzofurans (PCDFs) in biochars produced from three different feedstocks (softwood, wheat straw, and anaerobic digestate). Different scaled pyrolysis units (one batch and two continuous units) at two different temperatures (550 and 700 °C) were considered. The results revealed that the type of biomass had a significant influence on the PAH, oxy-PAH, and N-PAC content of the biochars. The configuration and type of the pyrolysis unit influenced only the wheat straw pyrolyzed at 550 °C. PCDDs and PCDFs occurred at very low levels in the biochars. In terms of PAH, PCDD, and PCDF content, the biochars assessed in this study represent a low risk to the environment, regardless of the temperature and type and size of the pyrolysis unit.

## Introduction

During pyrolysis, materials undergo thermochemical decomposition at temperatures above 300 °C in an oxygen-limited environment. The yield and chemical composition of the resulting pyrolysis products vary with the characteristics of the feedstock and the process variables, e.g., heating rate and residence time of the process (Mohan et al. 2006; Zhao et al. 2013). To date, the liquid and gas fraction has been the most analyzed pyrolysis product. However, biochar from lignocellulosic biomass has gained significant attention due to its properties and potential use in environmental and agricultural applications (e.g., as soil amendment and replacement for or supplement to activated carbon (Lehman and Joseph 2009)).

Thermal decomposition of biomass yields a complex mixture of condensable hydrocarbons, i.e., tar, which consists of single- to five-ring aromatics, phenolic compounds, and complex polycyclic aromatic hydrocarbons (PAHs) (Wolfesberger et al. 2009). The tar-like products are highly branched at moderate temperatures (~ 500 °C) (Pakdel and Roy 1991), but (in general) highly condensed and less oxygenated at high temperatures (> 700 °C) (Elliott 1986; Baker and Elliott 1986). Oxygenated-PAHs (oxy-PAHs) and nitrogen-containing heterocyclic polycyclic aromatic compounds (N-PACs), which typically occur as PAH co-pollutants in soils and groundwater (Lundstedt et al. 2003; Arp et al. 2014), display similar toxicity to PAHs (Andersson and Achten 2015). Therefore, oxy-PAHs should be considered for inclusion in biochar regulations. Oxy-PAHs may form through either biological, chemical or photo-oxidation (Andersson and Achten 2015), or catalytic transformation of PAHs (Nielsen et al. 1999), whereas N-PACs form via pyrolysis of lignocellulose materials and sewage (Britt et al. 2002). N-PACs have been reported from the pyrolysis of sewage sludge (Fullana et al. 2003). During thermochemical processes, PAHs (Weber et al. 2001) and oxy-PAHs (Hajizadeh et al. 2011) may also act as precursors for the formation of chlorinated aromatics, such as polychlorinated dibenzo-*p*-dioxins (PCDDs) and polychlorinated dibenzofurans (PCDFs).

Biochar intended for soil application must fulfill certain property- and composition-related requirements, to prevent harm to the ecosystem (Buss and Mašek 2014). Therefore, several guidelines with suggested threshold values for contaminants (including PAHs, PCDDs, or PCDFs) in biochar have been established, and the importance of contaminant analysis has been emphasized (British Biochar Foundation 2014; International Biochar Initiative 2015; European Biochar Foundation 2016).

Biochars with low concentration of contaminants may be achieved by selecting suitable feedstocks and controlling the operating conditions in the pyrolysis unit. Therefore, knowledge of the variation in different process variables associated with different pyrolysis units is essential for producing high-quality biochar. However, a relationship between the pyrolysis temperature and the concentration of PAH in biochar remains elusive (Freddo et al. 2012; Hale et al. 2012; Kloss et al. 2012; Devi and Saroha 2015). Buss et al. (2016a) have attributed this to a simultaneous increase in PAH formation and evaporation from the biochar with increasing temperature. The influence of the scale, reactor type, and configuration of the pyrolysis unit on the PAH concentration of the biochar remains unclear. In addition, the concentration of chlorinated organics in biochar has rarely been assessed (Hale et al. 2012; Wiedner et al. 2013), and to the best of our knowledge, the concentrations of oxy-PAH and N-PAC in biochars have yet to be reported.

In this study, we evaluate organic contaminants (PCDD, PCDF, PAH, oxy-PAH, and N-PAC) found in biochars from different biomass materials treated in different types of pyrolysis units. Three different feedstocks (softwood pellets, wheat straw pellets, and anaerobic digestate) were pyrolyzed at two different temperatures (550 and 700 °C), using three pyrolysis setups (one batch and two continuous units) of different scales.

## Materials and methods

### Feedstocks

The biochars were produced from three different types of biomass: commercial softwood (pine and spruce) pellets (Premium Puffin, Puffin Pellets), commercial wheat straw pellets (Agripellets Ltd.), and anaerobically digested sewage sludge (AD) from water-treatment works. The characterization of these feedstocks, proximate and ultimate analysis, are shown in Table 1.

**Table 1:** Proximate and ultimate analysis for the studied feedstocks

	Unit	Softwood	Wheat straw	Anaerobic digestate
Moisture	wt% (a.r.)	6.71 ± 0.03 (5)	7.22 ± 0.22 (5)	5.72 ± 0.27 (6)
Volatiles	wt% (d.b.)	83.6 ± 0.4 (5)	76.3 ± 0.5 (5)	64.6 ± 0.4 (6)
Fixed carbon	wt% (d.b.)	14.4 ± 0.4 (5)	16.6 ± 1.1 (5)	7.7 ± 0.3 (6)
Ash	wt% (d.b.)	1.1 ± 0.1 (5)	7.0 ± 0.3 (5)	27.8 ± 0.5 (6)
C	wt% (d.b.)	49.9 (2)	45.2 (2)	38.0 (2)
H	wt% (d.b.)	6.6 (2)	5.4 (2)	0.60 (2)
N	wt% (d.b.)	< 0.10 (2)	0.58 (2)	4.29 (2)

*wt*, weight; *a.r.*, as received; *d.b.*, dry basis; *n*, number of replicates

### Pyrolysis experiments

The pyrolysis experiments were conducted in three different pyrolysis reactors (one batch reactor and two continuous reactors) located at the UK Biochar Research Centre (UKBRC), University of Edinburgh. Key characteristics of the units are presented in Table 2 and further details can be found in the referenced articles.

**Table 2:** Characteristics of the pyrolysis reactors used in this study

Name	Operation mode	Temperature	Type of heating	Capacity	Carrier gas flow	Ref.
Stage I	Batch	Max ~ 1200 °C	Infrared furnace	30–50 g run^−1^	0.3 L min^−1^	Crombie et al. 2013
Stage II	Continuous—auger	Max ~ 850 °C	Electric split-tube furnace	500 g h^−1^	1 L min^−1^	Buss et al. 2016-b
Stage III	Continuous—rotary kiln	Max ~ 850 °C	Set of electric heaters	30–50 kg h^−1^	10 min^−1^	Buss et al. 2014

An overview of the performed pyrolysis experiments and the collected samples is provided in Table 3. In the two continuous reactors (stages II (Buss et al. 2016b) and III (Buss and Mašek 2014)), mean residence times of 20 min were applied for all materials. The residence time was estimated by establishing first the temperature profile of the biomass/char bed along the rotary kiln reactor (stage III, Table 2) as well as the residence time distribution of particles in the reactor. Based on this information, the corresponding heating rate experienced by particles in the reactor and their residence time at the peak temperature were calculated. Therefore, while the mean residence time of particles in continuous reactor was 20 min, the residence time at peak temperature was only between 5 and 10 min, depending on the material used. Thus, obtained parameters were then used as settings in the batch reactor to reproduce the conditions in the continuous pyrolysis unit as closely as possible. In the stage I reactor (Crombie et al. 2013), the retention time at the highest treatment temperature was adapted to reflect the retention times in the heated areas of the furnace of the continuous reactors. Therefore, softwood was exposed for 10 min (each) to temperatures of 550 and 700 °C, whereas wheat straw was exposed for 5 and 6 min, respectively.

**Table 3:** Overview of pyrolysis experiments. X denotes the samples that were analyzed for contaminants. In addition, the product yields for the stage I unit are shown with the number of replicates in parentheses

Feedstock		550 °C	Stage I			Stage II		Stage III	
			Yield (%) 550 °C	700 °C	Yield (%) 700 °C	550 °C	700 °C	550 °C	700 °C
Softwood	Biochar	X	21 ± 1.1 (4)	X	19 ± 0.5 (4)	X	X	X	X
	Liquid	X	46 ± 1.1	X	45 ± 0.9				
	Gas		33 ± 2.1		36 ± 0.8				
Wheat straw	Biochar	X	25 ± 0.5 (3)	X	23 ± 0.8 (3)	X	X	X	X
	Liquid	X	44 ± 0.3	X	44 ± 0.8				
	Gas		31 ± 0.7		33 ± 0.8				
Anaerobic digestate	Biochar	X	25 ± 1.5 (7)						
	Liquid	X	49 ± 1.3						
	Gas		26 ± 1.2						

### Sample extraction and cleanup

We determined the fraction of 16 EPA priority PAHs (US EPA 2015), 11 oxy-PAHs (Arp et al. 2014; Andersson and Achten 2015), and 4 N-PACs (Arp et al. 2014), PCDDs, and PCDFs (homolog sums and WHO_2005_-TEQ) (Van den Berg et al. 2006)) occurring in biochars and liquid samples. The target PAHs, oxy-PAHs, and N-PACs are listed in Table 4.

**Table 4:** List of analyzed PAH, oxy-PAH, and N-PAC and the number of aromatic rings (n_Ar_) in each structure

PAH	n_Ar_	Oxy-PAH	n_Ar_	N-PAC	n_Ar_
Naphthalene	2	1-Indanone	2	Quinoline	2
Acenaphthylene	3	1-Acenaphthenone	3	Benzo[h]quinoline	3
Acenaphthene		9-Fluorenone		Acridine	
Fluorene		Anthracene-9,10-dione		Carbazole	
Phenanthrene		2- Methylanthracene-9,10-dione			
Anthracene		Cyclopentaphenanthrenone	4		
Fluoranthene	4	Benzo[a]fluorenone			
Pyrene		Benz[de]anthracen-7-one			
Benzo[a]anthracene		Benz[a]anthracene-7,12-dione			
Chrysene		Naphthacene-5,12-dione			
Benzo[b]fluoranthene	5	Benzo[cd]pyren-6-one	5		
Benzo[k]fluoranthene					
Benzo[a]pyrene					
Dibenz[ah]anthracene					
Indeno[cd]pyrene	6				
Benzo[ghi]perylene					

n_Ar_: number of aromatic rings in each structure

The chars were extracted, at 150 °C, via pressurized liquid extraction (Dionex 350, Thermo Fisher Scientific, Waltham, USA) using toluene of analytical grade quality (Fluka, ≥ 99.7%), following the procedure outlined by Gao et al. (2015). The liquid fraction was extracted via liquid/liquid extraction using n-hexane. For PCDD and PCDF analysis, the extracts were all cleaned up using a multi-layer silica column followed by fractionation with an AX-21 carbon/celite column (see Liljelind et al. (2003) for further details of the method). For analysis of PAHs, oxy-PAHs, and N-PACs in the char samples, cleanup was conducted using open columns containing 5 g KOH-silica, eluted with dichloromethane. The clean extracts were concentrated to ~ 1 mL of toluene. The liquid samples were cleaned via the same procedure, using n-hexane (rather than dichloromethane) as the eluent. Further details of the method for PAH, oxy-PAH, and N-PAC analysis are provided elsewhere (Arp et al. 2014). All samples were single samples.

### Instrumental analysis

The analyses were all performed on a GC-HRMS–Hewlett-Packard 5890 gas chromatograph (Agilent Technology, Santa Clara, USA) coupled to an Autospec Ultima Mass Spectrometer (Waters Corporation, Milford, USA), using a DB5 column (60 m length, 0.32 mm internal diameter, 25 μm film thickness) (Agilent Technology, Santa Clara, USA). The main purpose of using HRMS was to make it possible to separate oxy-PAHs from PAHs, and the method used was previously described by Arp et al. (2014). The mass spectrometer was operated in electron impact ionization/selected ion-monitoring mode and analytes were quantified using the isotope dilution technique. PAHs, oxy-PAHs, and N-PACs were identified by comparing retention times to quantification congeners in the reference standard, while PCDD and PCDF were compared with published GC-MS chromatograms (Ryan et al. 1991; Bacher et al. 1992).

### QA/QC

All laboratory work was performed using validated methods, and laboratory blanks were extracted with the samples and treated as samples (i.e., single samples) throughout the cleanup process. The reported concentrations (signal-to-noise ratio: 10) were all higher than the limit of quantification (LOQ). For all reported concentrations, blank concentrations were below five times sample concentrations. Due to this cutoff, acenaphthylene was removed from the reported results due to analytical uncertainties.

## Results and discussion

### PAHs in the biochars

**Fig. 1:**
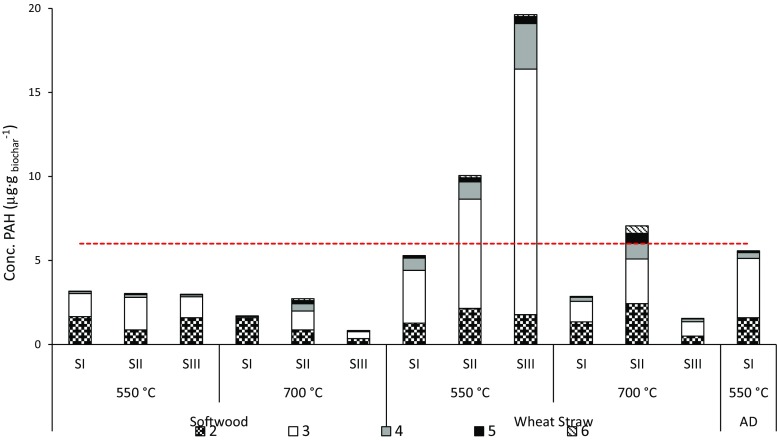
Total concentration and distribution of different-sized PAHs (numbers of rings) in each biochar. SI–SIII: size of the pyrolysis equipment (stage I, stage II, and stage III, respectively) and AD: anaerobic digestate. Red dashed line represents the range of lower-limit threshold values (set by IBI (International Biochar Initiative 2015)) for PAHs in biochar used in soil

The total PAH concentrations measured in the biochars varied considerably, from 0.82 to 19.6 μg g_biochar_^−1^, and seemed to be largely influenced by the feedstock type. This is particularly striking for concentrations in the three biochars produced at 550 °C (Fig. 1). For the same pyrolysis unit, the PAH concentrations of the wheat straw-derived biochars were (in some cases) almost seven times higher than those obtained from the softwood feedstock. The PAHs in biochars from all three feedstocks consisted mainly of two- and three-ring species, regardless of the pyrolysis temperature and unit scale. Furthermore, multi-ringed species, i.e., four- to six-ringed PAHs, occurred more abundantly in the wheat straw-derived biochar than in the other feedstocks. However, consistent with a previous study (Keiluweit et al. 2012), wheat straw-derived biochars produced at 700 °C contained higher concentrations of PAHs than those produced at 550 °C. The unit size had a substantial impact on PAH formation only for wheat straw pyrolyzed at 550 °C, where PAH formation was positively correlated with the unit size.

The International Biochar Initiative (IBI) guidelines have established threshold values, 6 and 300 μg g_biochar_^−1^, lower and upper limit, respectively, for the total PAH concentration of biochar (see Fig. 1) (International Biochar Initiative 2015). The PAH concentrations of the softwood-derived biochars were all less than the lower limit (6 μg g_biochar_^−1^), regardless of the pyrolysis temperature and the type and size of the unit. All biochars produced in this study meet the IBI PAH standards, and are thereby considered safe for use as soil amendments; softwood yielded the biochar with the lowest potential risk for PAH-related effects.

### Oxygenated-PAHs and N-PACs in the biochars

**Fig. 2:**
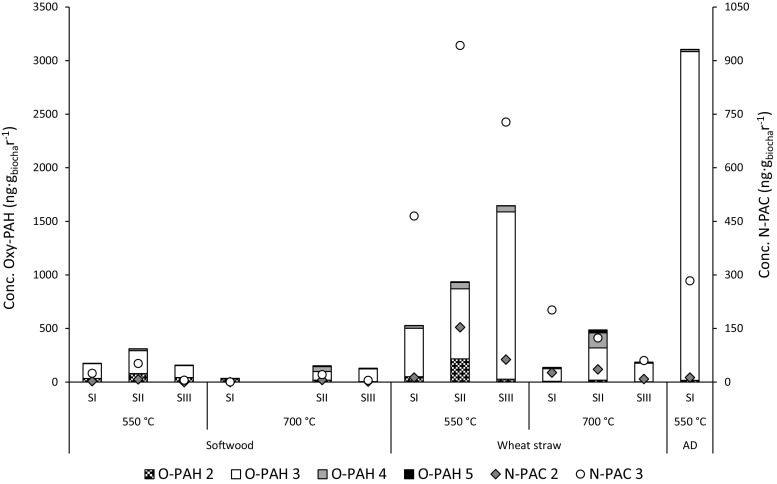
Concentrations (plotted on different scales) of oxy-PAH (columns) and N-PAC (bullets) in biochar from the different pyrolysis units. SI–SIII: size of the pyrolysis equipment (stage I, stage II, and stage III, respectively) and AD: anaerobic digestate

Oxy-PAHs and N-PACs occurred at detectable levels in all biochars, but the corresponding concentrations were lower than those of the PAHs (Fig. 2). The total oxy-PAH and N-PAC concentrations ranged from 34 to 3100 ng g_biochar_^−1^ and 0.4 to 477 ng g_biochar_^−1^, respectively. The oxy-PAHs and N-PACs consisted mainly of three-ringed species, and slightly higher concentrations of the multi-ringed oxy-PAH species were generated during wheat straw pyrolysis than during softwood pyrolysis. As in the case of PAHs, compared with the pyrolysis reactor size the feedstock exerted a larger influence on oxy-PAH formation. Furthermore, the reactor size had a substantial impact only on the oxy-PAH concentration in the biochar generated from wheat straw pyrolyzed at 550 °C. The highest concentration of oxy-PAH, which was more than 18 times higher and almost 6 times higher than those of softwood and wheat straw, respectively, was generated during stage 1 pyrolysis (at 550 °C) of anaerobic digestate. In this case, the elevated oxy-PAH concentrations are attributed to the composition of the digestate feedstock, which differs from those of the other two feedstocks.

The highest concentration (477 ng g_biochar_^−1^) of N-PACs occurred in wheat straw (rather than the anaerobic digestate) pyrolyzed at 550 °C (stage II), despite the anaerobic digestate having higher total N content than the wheat straw (Table 1). This possibly indicates that not total content, but actual N speciation (organic/inorganic) dictates the formation magnitude of the N-PAC species. Discussion on the toxicity of oxy-PAHs and N-PACs is ongoing, and therefore, threshold values for these compounds in biochar are lacking (Andersson and Achten 2015).

### Distribution of PAH, oxy-PAH, and N-PAC between biochar and the liquid fraction in stage I (SI)

**Fig. 3:**
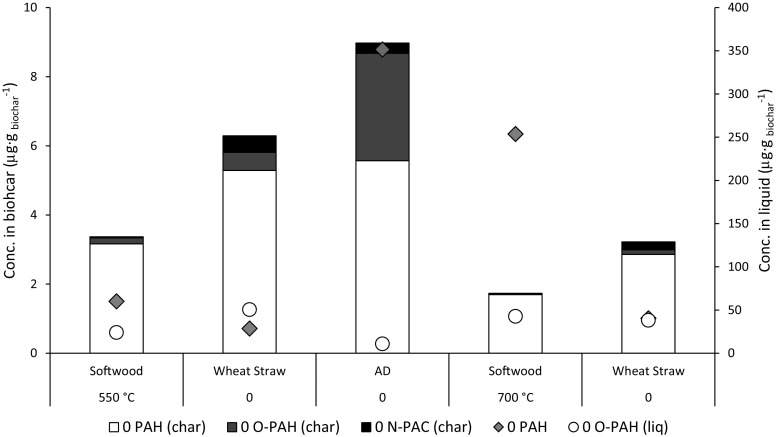
Distribution of PAH, O-PAH, and N-PAC in char, and PAH and O-PAH in the liquid fraction. N-PAC in the liquid fraction is not shown since the corresponding concentrations were below the limit of quantification (LOQ)

The liquid fraction, which was only collected from stage I (batch reactor, Table 2), constituted 44–49% of the total product yield of the pyrolysis process (Table 3). The distribution of PAH, oxy-PAH, and N-PAC between the biochar and liquid fraction showed that these products occurred mainly in the liquid fraction. In Fig. 3, the concentrations of PAH and oxy-PAH is shown. For example, the PAH concentration of the liquid fraction ranged from 28.6 to 351 μg g^−1^ while the concentrations of biochar were 1.7 to 5.6 μg g^−1^, making the concentration in the liquid 5–140 times larger than in the char. The distribution of the PAH and oxy-PAH in the liquid fraction tended to be lighter species, with less rings, compared to the char. All six-ringed PAHs in the liquid samples were below LOQ, N-PAC concentrations in the liquid fraction were compared to the PAH and oxy-PAH concentrations too low to be included in the Fig. 3, but except for wheat straw pyrolyzed at 550 °C, 80% or more of the total N-PAC were found in the liquid fraction. For the wheat straw sample, equal amounts were found in both liquid and char. Fagernäs et al. (2012) found that 62, 37, and only 0.6% of the PAHs occurred in the tars, gases, and the char, respectively. We did not measure the PAH concentration of the gases, but found that a considerably higher amount of PAHs occurred in the pyrolysis liquids than in the solids/char (PAH content of the solids: 0.3–9.3%). This demonstrates that PAH separation from char, via evaporation, is very effective. However, to prevent PAH deposition onto the biochar, contact between pyrolysis vapors (liquids and gases) and char in the very cold sections of the pyrolysis unit must be avoided.

Regardless of the operating conditions during the softwood and wheat straw runs, the PAH, oxy-PAH, and N-PAC content of the biochars decreased with increasing pyrolysis temperature, whereas the PAH content of the liquid fraction increased. These results suggest that although pyrolysis at 700 °C yields more PAHs (than pyrolysis at 550 °C), this process can generate biochars with lower levels of these potentially toxic compounds, as most PAHs evaporate from the char.

### Polychlorinated dibenzo*-p-*dioxins and furans

Consistent with a previous study (Wiedner et al. 2013), the results from this screening study showed that extractable polychlorinated aromatics species occur at almost negligible levels in the biochar matrix. Some PCDDs and PCDFs in the biochar, although detectable, were non-quantifiable (LOQ_PCDF_: 0.2 pg g_biochar_^−1^, LOQ_PCDD_: 0.3 pg g_biochar_^−1^). Monochlorinated dibenzofuran (MoCDF) was the only homolog that occurred with concentrations exceeding blank concentrations by some margin (defined as blank concentration times five), and this occurred in only 7 of the 13 biochars. Formation of MoCDF occurred more easily (and was therefore favored) at lower pyrolysis temperature and at larger pyrolysis unit (stage II and stage III) (data not shown), than at high pyrolysis temperatures and smaller scale. However, quantification of mono- to trichlorinated dioxins and furans in the liquid fraction was prevented by considerable matrix interference. Similarly, highly chlorinated congeners (hepta- and octachlorinated dioxins and furans) in the liquid fraction obtained from softwood and anaerobic digestate at 550 °C were non-quantifiable (LOQ 4 pg g_biochar_^−1^).

As the potential concentration of toxic PCDD and PCDF congeners in the biochars could not be calculated, approximate values were obtained by assigning to each congener a concentration equal to the LOQ values. Using this criterion, maximum concentrations of 0.6–0.9 pg_TEQ_ g_biobiochar_^−1^, which are slightly higher than TEQ concentrations reported for biochars generated from slowly pyrolyzed biomass (Hale et al. 2012), are expected for the toxic components of the biochars. The estimated worst-case TEQ concentrations are lower than the PCDD and PCDF threshold (17 pg_TEQ_ g_biochar_^−1^) established by IBI for biochars that will be used as soil amendment (International Biochar Initiative 2015). As in the case of PAHs, regardless of the temperature, configuration, or size of the pyrolysis unit, the biochars are expected to have low environmental impact when used for soil-improvement purposes.

Using LOQ, concentrations yielded an estimated worst-case TEQ concentration of ~ 1 pg_TEQ_ g_biochar_^−1^ for the liquid fraction.

## Conclusions

The influence of temperature and type/size of the pyrolysis unit on the concentration of four groups of toxic contaminants was evaluated in biochars from three different biomass feedstocks representing three major types of biomass, namely: forestry and agricultural residues, and organic waste. The results revealed that the type of biomass has a significant influence on the concentration of PAH, oxy-PAH, and N-PAC; the configuration and type/size of the pyrolysis unit have a significant effect only on wheat straw pyrolyzed at 550 °C.

This study represents the first-ever investigation where the content of oxy-PAH and N-PAC contaminants in biochars is determined. The results showed that in all cases this content is considerably lower than the PAH concentration. Regardless of the pyrolysis temperature, the PAH, oxy-PAH, and N-PAC concentrations were much higher in the liquid fraction compared to the char fractions. The PDCCs and PCDFs occurred at negligible levels in the studied biochars. Moreover, in terms of PAH, PCDD, and PCDF content, and regardless of the temperature and pyrolysis unit, the biochars are expected to have low negative environmental impact related to these contaminants when used for soil amendment. This demonstrates that biochar with extremely low content of organic pollutants can be produced from a range of materials and by using various technologies, which is an important milestone on the way to widespread biochar deployment and commercialization.
